# A Novel, Pan-PDE Inhibitor Exerts Anti-Fibrotic Effects in Human Lung Fibroblasts via Inhibition of TGF-β Signaling and Activation of cAMP/PKA Signaling

**DOI:** 10.3390/ijms21114008

**Published:** 2020-06-03

**Authors:** Katarzyna Wójcik-Pszczoła, Grażyna Chłoń-Rzepa, Agnieszka Jankowska, Marietta Ślusarczyk, Paweł E Ferdek, Agnieszka A Kusiak, Artur Świerczek, Krzysztof Pociecha, Paulina Koczurkiewicz-Adamczyk, Elżbieta Wyska, Elżbieta Pękala, Reinoud Gosens

**Affiliations:** 1 Department of Pharmaceutical Biochemistry, Faculty of Pharmacy, Jagiellonian University Medical College, Medyczna 9, 30-688 Kraków, Poland; paulina.koczurkiewicz@uj.edu.pl (P.K.-A.); elzbieta.pekala@uj.edu.pl (E.P.); 2 Department of Medicinal Chemistry, Faculty of Pharmacy, Jagiellonian University Medical College, Medyczna 9, 30-688 Kraków, Poland; mfchlon@cyf-kr.edu.pl (G.C.-R.); agnieszka.w.jankowska@doctoral.uj.edu.pl (A.J.); mari1106@o2.pl (M.Ś.); 3 Department of Cell Biology, Faculty of Biochemistry, Biophysics and Biotechnology, Jagiellonian University, Gronostajowa 7, 30-387 Kraków, Poland; pawel.ferdek@uj.edu.pl (P.E.F.); a.kusiak@student.uj.edu.pl (A.A.K.); 4 Department of Pharmacokinetics and Physical Pharmacy, Faculty of Pharmacy, Jagiellonian University Medical College, Medyczna 9, 30-688 Kraków, Poland; swierczek.artur@gmail.com (A.Ś.); k.pociecha@uj.edu.pl (K.P.); mfwyska@cyf-kr.edu.pl (E.W.); 5 Department of Molecular Pharmacology, University of Groningen, Antonius Deusinglaan 1, 9713 AV Groningen, The Netherlands; r.gosens@rug.nl

**Keywords:** asthma, COPD, myofibroblasts, airway remodeling, fibroblast-to-myofibroblast transition, TGF-β, phosphodiesterases, cAMP, TRPA1 channels, calcium influx

## Abstract

Phosphodiesterase (PDE) inhibitors are currently a widespread and extensively studied group of anti-inflammatory and anti-fibrotic compounds which may find use in the treatment of numerous lung diseases, including asthma and chronic obstructive pulmonary disease. Several PDE inhibitors are currently in clinical development, and some of them, e.g., roflumilast, are already recommended for clinical use. Due to numerous reports indicating that elevated intracellular cAMP levels may contribute to the alleviation of inflammation and airway fibrosis, new and effective PDE inhibitors are constantly being sought. Recently, a group of 7,8-disubstituted purine-2,6-dione derivatives, representing a novel and prominent pan-PDE inhibitors has been synthesized. Some of them were reported to modulate transient receptor potential ankyrin 1 (TRPA1) ion channels as well. In this study, we investigated the effect of selected derivatives (832—a pan-PDE inhibitor, 869—a TRPA1 modulator, and 145—a pan-PDE inhibitor and a weak TRPA1 modulator) on cellular responses related to airway remodeling using MRC-5 human lung fibroblasts. Compound 145 exerted the most considerable effect in limiting fibroblast to myofibroblasts transition (FMT) as well as proliferation, migration, and contraction. The effect of this compound appeared to depend mainly on its strong PDE inhibitory properties, and not on its effects on TRPA1 modulation. The strong anti-remodeling effects of 145 required activation of the cAMP/protein kinase A (PKA)/cAMP response element-binding protein (CREB) pathway leading to inhibition of transforming growth factor type β_1_ (TGF-β_1_) and Smad-dependent signaling in MRC-5 cells. These data suggest that the TGF-β pathway is a major target for PDE inhibitors leading to inhibitory effects on cell responses involved in airway remodeling. These potent, pan-PDE inhibitors from the group of 7,8-disubstituted purine-2,6-dione derivatives, thus represent promising anti-remodeling drug candidates for further research.

## 1. Introduction

Cyclic nucleotide phosphodiesterases (PDEs) are cellular enzymes responsible for the hydrolysis of phosphodiester bonds in two second messengers‘—adenosine 3′,5′-cyclic monophosphate (cAMP) or guanosine 3′,5′-cyclic monophosphate (cGMP). PDEs are grouped into 11 subfamilies (PDE1–PDE11) characterized by different substrate specificities: cAMP-specific (PDE4, PDE7, and PDE8), cGMP-specific (PDE5, PDE6, and PDE9) and both, cAMP and cGMP-specific (PDE1, PDE2, PDE3, PDE10, and PDE11) [1]. Therefore, the range of available inhibitors and their therapeutic potential is enormous [1]. Their potential therapeutic application covers disorders from heart failure and fertility, through Parkinson’s and Alzheimer’s disease, as well as depression and schizophrenia, to a wide range of inflammatory diseases [1]. Anti-inflammatory and anti-fibrotic properties of cAMP were demonstrated in different cellular models [2]. The cAMP was reported to be involved in regulating the function of both inflammatory cells as well as lung and bronchi structural cells in respiratory diseases, which inspired a growing interest in the potential therapeutic applications of PDE inhibitors [3,4]. A substantial body of evidence indicates that elevated cAMP levels promote airway smooth muscle cell (ASMC) relaxation and decrease ASMC/lung fibroblast proliferation, migration, and the ability to synthesize extracellular matrix (ECM) proteins, as well as reduce lung fibroblast to myofibroblast transition (FMT) [2,3,5]. Also, cGMP inhibitors are currently receiving increasingly more interest [1,4,6], owing to the facts that (1) cigarette smoking was associated with a decreased level of guanylyl cyclase [7] and (2) that stimulation of this cGMP-producing enzyme can reduce oxidative stress in chronic obstructive pulmonary disease (COPD) [8]. Additionally, it is known that smoking can induce PDE3 and PDE4 expression in the lungs [9], which not only indicates PDEs involvement in COPD but is also good further evidence confirming the validity of using PDE inhibitors in the treatment of lung diseases.

The first non-selective PDE inhibitor used in asthma therapy was theophylline (1,3-dimethylpurine-2,6-dione), a compound that belongs to the group of methylxanthines [10]. However, a number of side effects often limit its therapeutic application. Recently, this and other naturally occurring methylxanthines were proposed as a potential treatments for pediatric respiratory tract diseases [11]. Since PDE1, PDE3, PDE4, PDE5, and PDE7 are most closely associated with the pathogenesis of asthma or COPD [1,3], there has been a need for more selective inhibitors suitable for targeting isoforms of these PDE families. The aim of this approach is not only to achieve a more precise blockade of these enzymes but also to reduce the side effects of non-selective PDE inhibitors. Several inhibitors have undergone clinical trials, and some of them, such as roflumilast (a PDE4 inhibitor), are currently used in the clinic [12]. Another example is the inhaled PDE4 inhibitor, CHF6001 currently in phase II clinical trials, which also exhibits preferential anti-inflammatory properties in COPD [13]. However, since the efficacy of PDE inhibitors has not been markedly improved by the increased selectivity toward a specific PDE, the focus of the research was moved towards dual or pan-PDE inhibitors. Pan-PDE inhibitors represent compounds that are able to inhibit various isoforms within different PDE classes. Another reason why such inhibitors could be desirable is the cell-specific expression and compartmentalized intracellular localization of individual PDEs in the cell, which could account for the synergistic effects of targeting multiple subtypes simultaneously [14]. The reason for testing PDE inhibitors with a broader spectrum of activity also lies in the fact that the expression of individual PDEs may change in response to irritants to which patients are exposed, or those that cause asthma or COPD, e.g., tobacco smoke [9]. Pan-PDE inhibitors may provide a more effective therapeutic approach than selectively acting agents, mainly through their improved spectrum of pharmacological activity [1,4]. Enhanced therapeutic potential of pan-PDE inhibitors is expected to result from their effect on a number of signaling pathways involved in the development of asthma and other lung or bronchial diseases [1,3].

Besides the above-mentioned PDEs, transient receptor potential ankyrin 1 (TRPA1) channels have recently been proposed to contribute to the pathogenesis of asthma and COPD [15]. These non-selective calcium-permeable channels have been implicated in allergic reactions, including the late allergic response, airway hyperresponsiveness and bronchoconstriction, neurogenic inflammation, and cough; these receptors were also suggested to function as toxicant sensors [16,17]. TRPA1 expression was described in both immune and lung structural cells, including epithelial cells, smooth muscle cells, and fibroblasts, as well as sensory neurons. They can be activated by many different irritants, e.g., allyl isothiocyanate, allicin, or acrolein as well as pro-inflammatory mediators, including histamine, prostaglandins, or bradykinin [15,16,17,18]. Therefore, it is believed that TRPA1 antagonists may serve as a promising therapeutic alternative for lung diseases [17,18].

**Figure 1:**
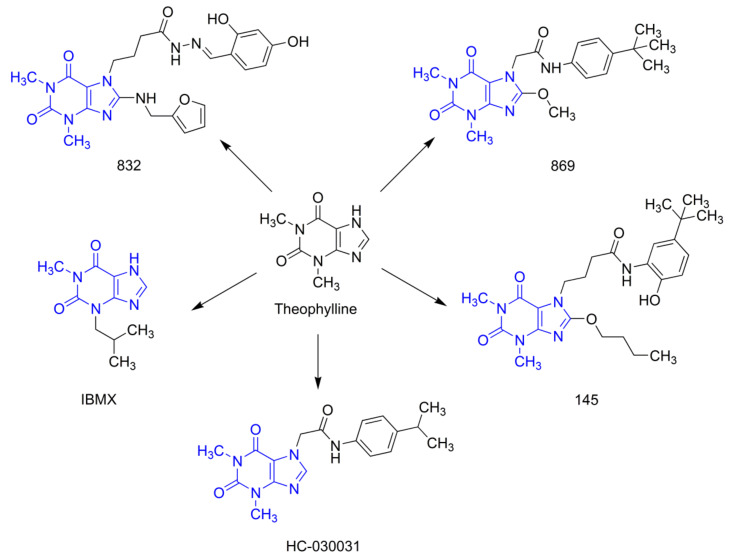
Chemical structures of 832, 869, 145, and reference compounds, IBMX and HC-030031, as theophylline derivatives.

Our earlier studies revealed that, compared to selective PDE4 inhibitors (roflumilast or cilomilast), pan-PDE inhibitors might provide better inhibition of the transforming growth factor type β_1_ (TGF-β_1_)-induced ASMC remodeling [19]. We have also reported that some of the recently synthesized 7,8-disubstituted purine-2,6-dione derivatives, in addition to being pan-selective PDE inhibitors, can interact with TRPA1 ion channels [20,21]. In this study, we selected three 7,8-disubstituted purine-2,6-dione derivatives (Figure 1): 832 (a pan-PDE inhibitor), 869 (a TRPA1 modulator), and 145 (a pan-PDE inhibitor and a TRPA1 modulator) and evaluated their ability to limit profibrotic responses of lung fibroblasts. For this purpose, we used TGF-β_1_ or fetal bovine serum (FBS)-activated MRC-5 cells and investigated proliferation, migration, contraction, expression of profibrotic genes, and phenotype transition into myofibroblasts under the influence of these 7,8-disubstituted purine-2,6-dione derivatives. Our goal was also to verify the TRPA1 contribution in the potential anti-fibrotic effects of 7,8-disubstituted purine-2,6-dione derivatives.

## 2. Results

### 2.1. Compounds 832 and 145 but not 869 are Prominent, Pan-PDE Inhibitors

Our previous studies revealed that both amide and hydrazide 7,8-disubstituted purine-2,6-dione derivatives represent a group of strong and non-selective PDE inhibitors [19,20,21,22]. This and previous experiments allowed us to determine the activity of the studied compounds against PDE1B, 3A, 4B, 4D, and 7A (Table 1). For most isoforms, the activity of 832 and 145 was much better than that of a non-selective, reference inhibitor, IBMX. To better understand the activity profile of 832, 869, and 145, we have expanded our research to include other isoforms of individual PDEs, particularly those involved in the pathogenesis of bronchial asthma. Luminescence analysis with human recombinant PDEs (recently a rapid mass spectrometric determination of AMP and cAMP level has been designed [23]) showed that 832 and 145 effectively inhibit also PDE1C, 3B, and 4A with IC_50_ values of 89.18 µM, 0.42 µM, 4.75 µM, and 48.52 µM, 2.05 µM, 1.59 µM, respectively (Table 1). Similar to IBMX, compound 832 was active against PDE1A. Interestingly, 145 was the only compound in the study group capable of inhibiting PDE8A, with an IC_50_ value of 73.18 µM (Table 1). In contrast to 832 and 145, compound 869 did not show significant activity as a PDE inhibitor (Table 1).

### 2.2. Compound 869 is a Representative Antagonist of the TRPA1 Channel

Our previous reports indicated that some of the 7,8-disubstituted purine-2,6-dione derivatives, in addition to PDE inhibition may also modulate TRPA1 channel activity [20]. Therefore, we determined the ability of 832, 869, and 145 to inhibit and activate the TRPA1 channel and compared the obtained results with reference compounds; a known TRPA1 channel antagonist—HC-030031 and a known TRPA1 channel agonist—ASP 7663. Out of all the studied compounds, only 869 showed strong TRPA1 channel antagonism, comparable to the reference HC-030031 (Table 2). It inhibited the TRPA1 channel activity by 74.9% and 98.7% at 10 µM and 50 µM, respectively (Table 2). The two other investigated compounds at best showed only a weak modulation of the TRPA1 channel (less than 25% of control) (Table 2).

### 2.3. 7,8-Disubstituted Purine-2,6-dione Derivatives Selectively Affect Activated Lung Fibroblasts’ Proliferation and Migration

**Figure 2:**
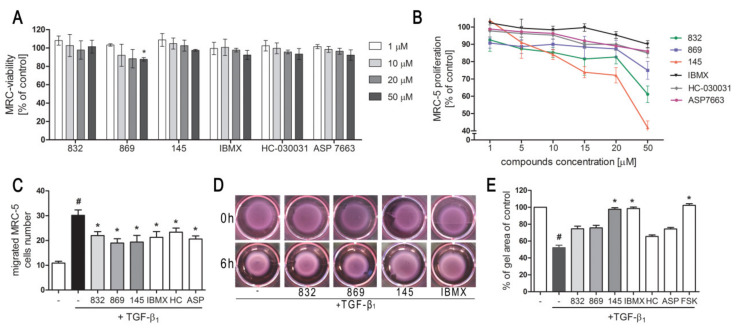
Compounds 832, 869, and 145 disturb FBS or TGF-β_1_-induced MRC-5 cells remodeling phenotype. (**A**) Lung fibroblasts were cultured in growing concentrations of 832, 869, 145, IBMX, HC-030031, and ASP7663 (1–50 µM) for 48 h. A viability assay was performed with CYTO-Tox One fluorescent kit according to the manufactures’ protocol. Values represent viable cells (% mean ± S.E.M.) in reference to controls; (**B**) Lung fibroblasts were cultured for 24 h and then serum-deprived for 72 h. MRC-5 proliferation in the growing concentrations of the studied compounds (1–50 µM) was determined using the alamarBlue^®^ assay after 48 h of incubation in the presence of FBS; *n* = 6. (**C**) Lung fibroblasts migration in response to TGF-β_1_ (5 ng/mL) was assessed after 24 h incubation with the studied compounds (10 µM). MRC-5 were stained with crystal violet, and any migrated cells were counted in 10 randomly selected fields of view. (**D**, **E**) MRC-5 contraction was determined after 1 h pre-incubation of collagen gel lattices in the presence of the studied compounds (0 h) and 6 h exposure to TGF-β_1_. (**D**) Representative pictures of collagen gel lattices. (**E**) Quantification of the collagen gel area after a 6-h long incubation in the presence of the studied compounds and TGF-β_1_. All values represent the mean (± S.E.M.). The results were considered statistically significant at the *p* level of 0.05 against the control (#) and TGF-β_1_ (*).

We first determined whether 832, 869, and 145 affect MRC-5 lung fibroblast viability. We exposed the cells to increasing concentrations (1–50 µM) of the study compounds or to reference controls (IBMX, HC-030031, and ASP 7663), and found that none of the tested compounds decreased the initial viability of MRC-5 cells (Figure 2A). To investigate the ability of 832, 869, and 145 to limit the proliferative and profibrotic responses of lung fibroblasts, we used two stimuli for the fibroblast activation: FBS (for proliferation) or TGF-β_1_ (for profibrotic responses). The selected 7,8-disubstituted purine-2,6-dione derivatives had differential activity in inhibiting FBS-induced lung fibroblast proliferation. Compound 832, 869, and 145 reduced MRC-5 proliferation more effectively than the reference compounds used in the study. At a lower concentration range (1–20 µM), 145 exerted the best anti-proliferative activity against FBS-induced MRC-5 cells, whereas for 832 and 869, this effect was much less prominent (Figure 2B, Table S1). Interestingly, virtually no change in the lung fibroblast proliferation rate was observed at lower concentrations of the TRPA1 channel agonist/antagonist. At the highest concentration applied (50 µM), FBS-induced MRC-5 proliferation rate was inhibited by 39%, 25%, and 58%, in the presence of 832, 869 and 145, respectively; while for IBMX, HC-030031, and ASP 7663 these values were 10%, 15%, and 14%, respectively (Figure 2B). In further analyses, cells were treated with compounds at 10 µM, because this concentration was both non-toxic and did not decrease the MRC-5 cell proliferation rate. As for TGF-β_1_-induced MRC-5 migration, all compounds, as well as the reference compounds, showed similar activity and slightly but statistically significantly reduced the ability of lung fibroblasts to move throughout the Transwell pores (Figure 2C). In turn, the differential effects of 7,8-disubstituted purine-2,6-dione derivatives were observed in the collagen gel contraction assay. As demonstrated in Figure 2D,E, the size of the collagen gel lattices after TGF-β_1_ and 832 or 869 increased only slightly compared to TGF-β_1_ administered alone. Compound 145 and IBMX had a different effect and restored the size of collagen gel lattices to that of the untreated control (Figure 2D). Forskolin, a cAMP activator, showed similar properties as 145 or IBMX, suggesting that an increased cAMP level may be responsible for the reversal of TGF-β_1_-induced collagen gel contraction (Figure 2E). At the same time, no effect of the TRPA1 channel agonist/antagonist on TGF-β_1_-induced lung fibroblast contraction was observed (Figure 2E).

### 2.4. Compound 145 Significantly Limits TGF-β_1_-Induced Lung Fibroblast to Myofibroblast Transition

**Figure 3:**
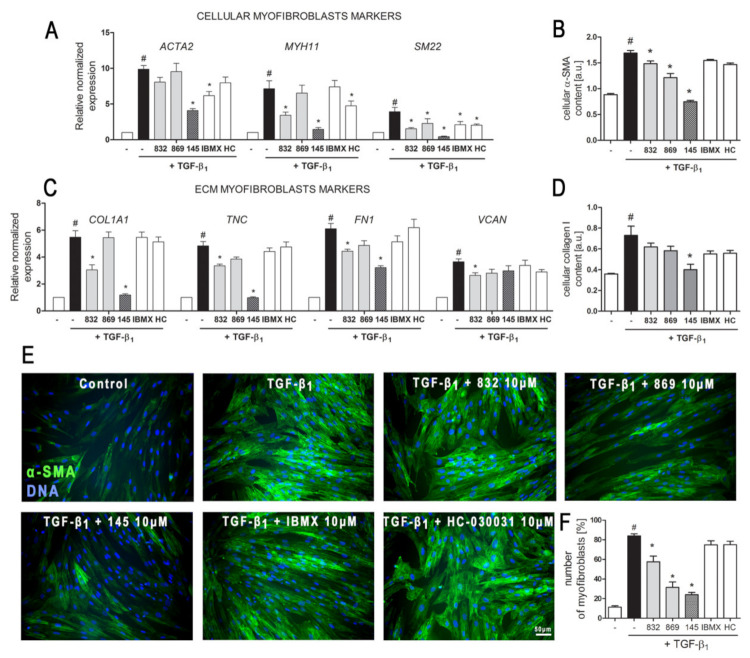
Compound 145 significantly reduced TGF-β_1_-induced MRC-5 transition into myofibroblasts. MRC-5 were pre-incubated for 1 h with the studied compounds (10 µM) and then cultured for 24 h (**A**, **C**) or 48 h (**B**, **D**–**F**) in TGF-β_1_ (5 ng/mL). (**A**, **C**) qPCR was carried out to analyze *ACTA2, MYH11, SM22, COL1A1, TNC, FN1*, and *VCAN* transcript levels. Samples were run three times in duplicates. Cellular α-smooth muscle actin (α-SMA) (**B**) and collagen I (**D**) protein level was determined by in-cell ELISA; *n* = 8. (**E**) To visualize α-SMA positive stress fibers, MRC-5 were fixed, permeabilized, blocked, and labeled with anti-α-SMA antibody, followed by Alexa Fluor 488 conjugated antibody, nuclei were stained with Hoechst 33342 dye. Microphotographs were taken using a Leica DMiL LED Fluo microscope, 40× objective, bar = 50 µm. (**F**) Myofibroblasts (MRC-5 positive for α-SMA) were counted in 20 randomly selected fields of view and expressed as a percentage of the entire MRC-5 population. Each bar represents the mean value (± S.E.M.). The results were considered statistically significant at the *p* level of 0.05, against control (#) and TGF-β_1_ (*).

The demonstrated properties of 7,8-disubstituted purine-2,6-dione derivatives prompted us to check whether 832, 869, and 145 may affect the TGF-β_1_-induced phenotype switch of lung fibroblasts into myofibroblasts. Transcriptional analysis of myofibroblast markers in MRC-5 cells, cultured in the presence of TGF-β_1_ and 832, 869, or 145, revealed that studied 7,8-disubstituted purine-2,6-dione derivatives exert different effects on the expression of target genes (Figure 3A,C).

The expression of all the analyzed myofibroblast markers: *ACTA2*, *MYH11*, *SM22*, *COL1A1*, *TNC*, *FN1*, and *VCAN* was significantly increased after activation with TGF-β_1_ (Figure 3A,C). In the 7,8-disubstituted purine-2,6-dione derivatives group, 145 showed the highest activity in reducing TGF-β_1_-induced myofibroblast gene expression and caused a two-fold, five-fold, nine-fold, four-fold, and five-fold decrease in the expression of *ACTA2*, *MYH11*, *SM22*, *COL1A1*, and *TNC*, respectively (compared to TGF-β_1_ administration alone) (Figure 3A,C). Compounds 832 and 869 showed slightly weaker effects on TGF-β_1_-induced expression of myofibroblast markers. The results of the transcriptional analysis were also confirmed at the protein level (Figure 3B,D). 832, 869, and 145 selectively reduced TGF-β_1_-induced cellular content of α-SMA and collagen I in lung fibroblasts. Also, in this case, compound 145 exhibited the most prominent properties and significantly reduced the levels of both proteins in TGF-β_1_-treated cells (Figure 3B,D). Transcriptional and protein analysis showed that our PDE inhibitors more efficiently diminished the TGF-β_1_-induced myofibroblast phenotype in MRC-5 cells than the non-selective, reference inhibitor, IBMX (Figure 3A–F). Since the expression and synthesis of α-SMA actin, as well as its incorporation into stress fibers is one of the main markers of fibroblast to myofibroblast transition, we also performed immunocytochemical staining of fibroblasts cultured in the presence of TGF-β_1_ and 832, 869, or 145 (Figure 3E). The number of myofibroblasts (MRC-5 with α-SMA-positive stress fibers) in cultures decreased in the presence of 832, 869, or 145, and the most significant decrease occurred with compound 145 (Figure 3F). Interestingly, the analyses did not show any significant effect for the TRPA1 channel antagonist, HC-030031, on TGF-β_1_-induced lung fibroblast to myofibroblast transition (Figure 3A–F).

### 2.5. Both Inhibition and Activation of TRPA1 Channels Affect Ca^2+^ Signals in MRC-5

**Figure 4:**
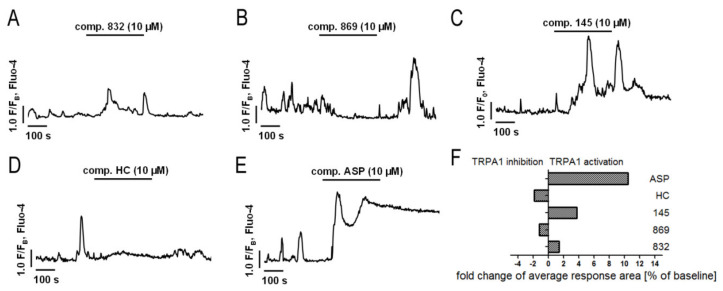
Both inhibition and activation of TRPA1 channels affect Ca^2+^ signals in MRC-5 cells. Traces show representative Ca^2+^ responses recorded in MRC-5, before and after acute application of one of the investigated compounds: (**A**) 832 (10 µM), *n* = 22; (**B**) 869 (10 µM), *n* = 23; (**C**) 145 (10 µM), *n* = 28; (**D**) HC-030031 (10 µM), *n* = 25; (**E**) ASP7663 (10 µM), *n* = 23. (**F**) Fold change of TRPA1 inhibition or activation after an acute application of investigated compounds.

TRPA1 is an ion channel within the plasma membrane, permeable for Ca^2+^. Since the tested compounds had very different effects on TRPA1 channel inhibition or activation, we decided to check whether they also affect the intracellular Ca^2+^ homeostasis. Analysis of spontaneous Ca^2+^ signals in MRC-5 revealed that out of the studied compounds, only 869 exhibited similar properties to the reference antagonist—HC-030031—and inhibited Ca^2+^ influx in lung fibroblasts (Figure 4). The other compounds triggered Ca^2+^ responses in MRC-5, although to a lesser extent than the reference agonist used in the analysis—ASP 7663. Compounds 832 and 145 caused a two-fold and four-fold increase in the average response area, respectively, while responses induced by ASP 7663 triggered, on average, a 10-fold elevation of intracellular Ca^2+^ over the baseline levels (Figure 4). The activation of TRPA1 by compound 145 at the concentration of 10 µM was much less prominent than that by ASP 7663. As opposed to ASP 7663, which caused sustained and prolonged increases in intracellular Ca^2+^, compound 145 triggered less pronounced oscillatory type Ca^2+^ signals.

### 2.6. Modulation of TRPA1 Ion Channel Does Not Affect Compound 145 Anti-Fibrotic Properties

**Figure 5:**
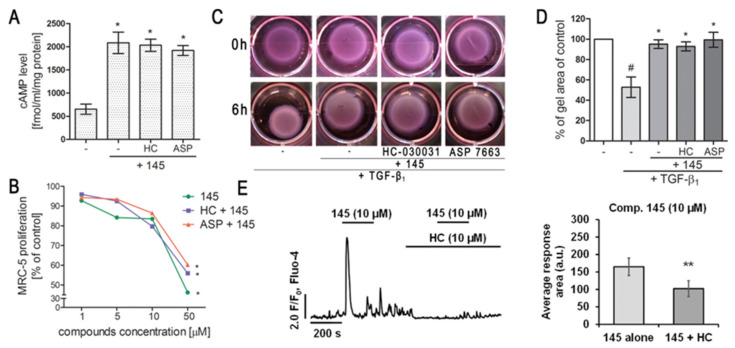
Modulation of TRPA1 channel activity does not affect the anti-fibrotic properties of compound 145 in MRC-5 cells. (**A**) Lung fibroblasts were cultured for 24 h and then serum-deprived for 2 h. The cAMP concentration (fmol/mL/mg of protein; *n* = 4) was measured after 30 min of pre-incubation with HC-030031 (10 µM) or ASP7663 (10 µM) followed by 30 min of incubation with 145. (**B**) Lung fibroblasts were cultured for 24 h and then serum-deprived for 72 h. MRC-5 proliferation in the growing concentrations of the studied compounds (1–50 µM) was determined using alamarBlue^®^ assay after 48 h of incubation in the presence of FBS; *n* = 6. (**C**, **D**) MRC-5 contraction was determined after 30 min of pre-incubation with HC-030031 (10 µM) or ASP7663 (10 µM) followed by 30 min of incubation with 145 and 6 h exposure to TGF-β_1_. (**C**) Representative pictures of collagen gel lattices taken at the beginning of the experiment (0 h) and after 6 h incubation. (**D**) Quantification of collagen gel area after a 6 h incubation in the presence of study compounds and TGF-β_1_. Each bar represents the mean (± S.E.M.) (**E**) MRC-5 cells were treated for 200 s with 145 (10 µM) and then again with 145 (10 µM) in the presence of HC-030031 (10 µM); representative trace (left); bar charts showing average Ca^2+^ increase above the baseline recorded for 200 s during the first and the second application of 145 (*n* = 30, right). The results were considered statistically significant at the *p* level of 0.05 (*) or 0.01 (**).

Since compound 145 showed the most promising anti-fibrotic properties without triggering an excessive Ca^2+^ influx in MRC-5, we decided to assess the overall TRPA1 component in the observed effect. To achieve this, we either blocked or activated TRPA1 in MRC-5 by preincubation with HC-030031 or ASP 7663, respectively, and then exposed the cells to 145. Neither the TRPA1 agonist nor the antagonist caused any significant changes in cAMP levels in lung fibroblasts obtained after incubation with compound 145 (Figure 5A). Moreover, compared to compound 145 alone, neither of the aforementioned TRPA1 modulators affected the FBS-induced lung fibroblast proliferation rate (Figure 5B, Table S2). Given that our experiments revealed that 145 is very efficient at restoring the TGF-β_1_-induced collagen gel contraction (Figure 2D,E), we decided to check whether this feature is dependent on TRPA1. Preincubation of MRC-5 with HC-030031 or ASP 7663, followed by the application of 145, did not result in the appearance of significant differences in the size of the collagen gel lattices compared to the treatment with 145 alone (Figure 5C,D). At the same time, compound 145 failed to induce Ca^2+^ responses under TRPA1 inhibition by HC-030031 in lung fibroblasts (Figure 5E).

### 2.7. Compound 145 Attenuates Smad-2 Phosphorylation in TGF-β_1_-Induced Lung Fibroblasts

**Figure 6:**
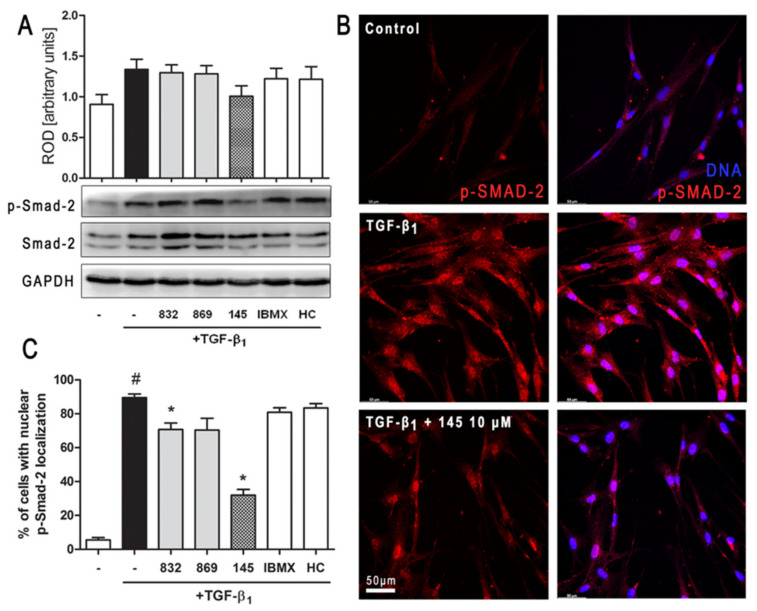
Compound 145 limits TGF-β_1_-induced Smad-2 phosphorylation and nuclear translocation in MRC-5 cells. Lung fibroblasts were pre-incubated with the studied compounds (10 µM) for 1 h, and then TGF-β_1_ (5 ng/mL) was added for 1 h. (**A**) Representative immunoblot and densitometry analysis of p-Smad-2 in MRC-5 (*n* = 4). The relative optical density (ROD) signal was normalized to the GAPDH control level. Each bar represents the mean value (± S.E.M.). (**B**) Immunofluorescence staining for p-Smad-2 protein: MRC-5 were fixed, permeabilized, blocked, and incubated with anti-p-Smad-2 antibody followed by Alexa Fluor 546 conjugated antibody, nuclei were stained with the Hoechst 33342 dye. Microphotographs were taken using Leica DMi8 microscope with Thunder Imaging System, 40× objective, bar = 50 µm. (**C**) Cells exhibiting strong nuclear p-Smad-2 signals were counted in 20 randomly selected fields of view and expressed as a percentage fraction in the entire MRC-5 population. The results were considered statistically significant at the *p* level of 0.05, against the control (#) and TGF-β_1_ (*) conditions.

Intracellular Smad proteins are one of the main effectors of TGF-β_1_ and constitute an essential part of its canonical signaling pathway. To investigate the contribution of Smad-2 to the effect of 145 in TGF-β_1_-induced lung fibroblasts, we checked the level of phosho-Smad-2. Immunoblotting revealed that Smad-2 phosphorylation induced by TGF-β_1_ was reduced by 145 (Figure 6A). Simultaneously, no differences in the TGF-β_1_-induced Smad-2 phosphorylation were present after incubation with 832, 869, IBMX, and HC-030031 (Figure 6A). The fraction of fibroblasts with nuclear localization of p-Smad-2 was significantly elevated in TGF-β_1_-induced MRC-5 (Figure 6B,C). While a slight decrease in nuclear localization of p-Smad-2 occurred in the cells treated with 832, compound 145 caused a very marked decrease of TGF-β_1_-induced nuclear localization of p-Smad-2 (a 57% decrease compared to TGF-β_1_ alone) (Figure 6C).

### 2.8. Compound 145 Activates cAMP/PKA/CREB Dependent Signaling in TGF-β_1_-Induced Lung Fibroblasts

**Figure 7:**
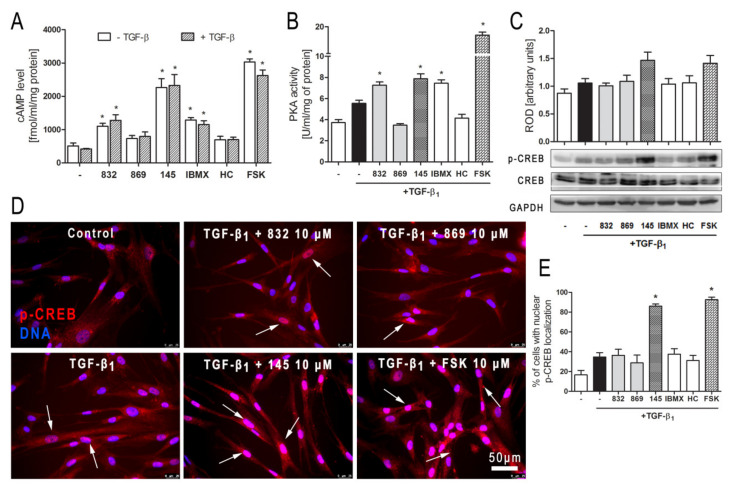
Compound 145 activates cAMP/PKA/CREB dependent pathway in TGF-β_1_-induced MRC-5 cells. (**A**) Lung fibroblasts were cultured for 24 h and then serum-deprived for 2 h. The cAMP level was measured after 30 min of incubation with the studied compounds (10 µM) and TGF-β_1_ (5 ng/mL). Each bar represents the mean (± S.E.M.) The cAMP concentration (fmol/mL/mg of protein; *n* = 4). (**B**) Protein kinase A activity in MRC-5 was determined after 30 min of incubation with the studied compounds (10 µM), and 30 min of incubation with TGF-β_1_ (*n* = 4). (**C**) Representative immunoblot and densitometry analysis of p-CREB in MRC-5 (*n* = 4). The ROD signal was normalized to the GAPDH control level. Each bar represents the mean value (± S.E.M.). (**D**) Immunofluorescence staining for p-CREB protein: MRC-5 were fixed, permeabilized, blocked, and incubated with anti-p-CREB antibody followed by an Alexa Fluor 546 conjugated antibody, nuclei were stained with Hoechst 33342 dye. Microphotographs were taken using a Leica DMi8 microscope, 40× objective, bar = 50 µm. (**E**) Cells exhibiting co-localization of p-CREB and nuclear signal were counted in 20 randomly selected fields of view and expressed as a percentage fraction in the entire MRC-5 population. The results were considered statistically significant at the *p* level of 0.05, against control (*).

In order to clarify further molecular mechanisms underlying the anti-fibrotic action of 145 on TGF-β_1_-induced human lung fibroblasts, we analyzed the cAMP/PKA/CREB pathway, which represents the main signaling pathway for compounds characterized as PDE inhibitors. Firstly, we determined the level of cAMP in MRC-5 cells exposed to selected 7,8-disubstituted purine-2,6-dione derivatives. Compound 145 showed the best activity and increased cAMP levels in lung fibroblasts from 508.87 fmol/mL/mg of protein in the control to 2262.07 fmol/mL/mg of protein (Figure 7A). The effects of other studied compounds were somewhat weaker, but still significant. For 832 and IBMX, a 2.16-fold and 2.53-fold increase in cAMP level, respectively, was observed (Figure 7A). The level of cAMP achieved in 145-treated fibroblasts (4.5-fold increase when compared to the control) was similar to that obtained for the known cAMP activator—forskolin (5.7-fold increase when compared to the control). The presence of TGF-β_1_ did not influence the cAMP levels in MRC-5 cells (Figure 7A). Protein kinase A activity in TGF-β_1_-induced fibroblasts correlated with the recorded cAMP levels and was the highest in forskolin treated cells and slightly lower in 145, IBMX, and 832-treated MRC-5 cells (Figure 7B). There was no significant change in the activity of this kinase in TGF-β_1_-induced cells incubated in the presence of HC-030031 or 869. The CREB phosphorylation data were in line with the presented above. CREB phosphorylation occurs as a result of elevated cAMP levels and increased PKA activity (Figure 7C). An increased CREB phosphorylation was found in TGF-β_1_-induced MRC-5 cultured in the presence of 145 or forskolin, whereas the other compounds had no effect on this process (Figure 7C). Also, phosphorylated CREB translocation to the nucleus was significantly more prominent in TGF-β_1_-induced MRC-5 cultures exposed to 145 and forskolin (Figure 7D). The fraction of MRC-5 cells with nuclear p-CREB localization was elevated by 70% and 76% in the presence of 145 and forskolin, respectively (Figure 7E). Compound 832, 869, IBMX, and HC-030031 caused only a slight increase in the percentage of fibroblasts exhibiting the nuclear localization of phosphorylated CREB (Figure 7D,E).

## 3. Discussion

Tissue remodeling is a widespread pathological process in which a number of structural changes occur in a tissue or organ that impairs its normal physiological functions [24,25,26]. In the lungs or bronchi, remodeling in asthma, COPD and lung fibrosis is often associated with the imbalance in ECM protein turnover, disturbances that occur in the structure and functions of the epithelial layer, smooth muscle cell hyperplasia and hypertrophy, and increased levels of tissue myofibroblasts, predominantly as a result of fibroblast to myofibroblast transition [27,28]. Therefore, in the search for new anti-fibrotic drugs, particular attention should be paid to pleiotropic compounds that affect various cellular processes associated with remodeling. We have recently shown novel PDE inhibitors from the group of 7,8-disubstituted purine-2,6-dione derivatives as potent modulators of ASMC remodeling [19]. Here we demonstrated for the first time that a strong pan-PDE inhibitor from this group, compound 145 can also exert anti-fibrotic effects in TGF-β_1_-induced human lung fibroblasts via limiting FMT.

A number of studies have earlier demonstrated FMT-limiting properties of cAMP elevating agents, such as PDE inhibitors or activators of soluble adenylyl cyclase [29,30,31]. The potential benefits of using theophylline as an anti-inflammatory drug in the treatment of asthma have long been known, but it was only a few years ago when Yano et al. reported the anti-FMT effects of this substance in the lung [32]. In turn, we demonstrated that other methylxanthines, i.e., pentoxifylline or lisofylline, can also reduce FMT in bronchial fibroblasts isolated from asthmatics [33]. Other studies revealed that selective inhibitors, particularly those inhibiting PDE4, such as rolipram or roflumilast, also show anti-FMT effects [31,34,35].

**Figure 8:**
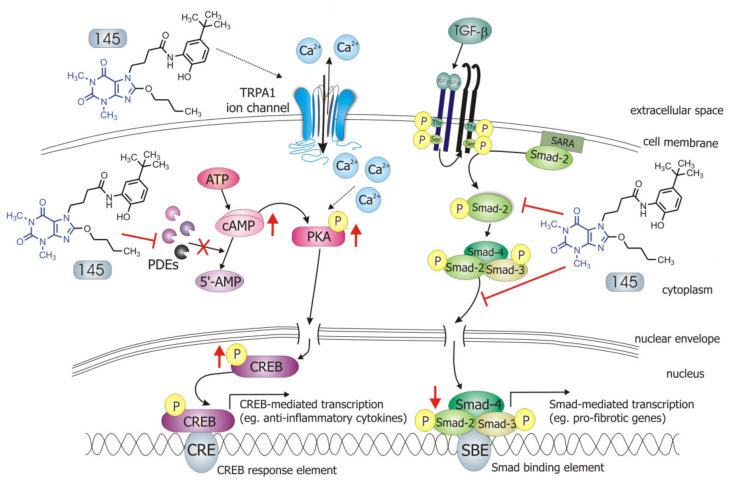
Compound 145 interaction with TGF-β_1_-induced MRC-5 cells and possible targets related to its anti-fibrotic effects. Abbreviations: ATP—adenosine triphosphate; 5′-AMP—5′-adenosine monophosphate; CRE—CREB response element; SBE—Smad binding element, SARA—Smad anchor for receptor activation

Our recently designed and synthesized 7,8-disubstituted purine-2,6-dione derivatives fill very well the gap between inhibitors highly selective for a single isoenzyme, and non-selective and weak non-specific inhibitors. Both our previous [19] and current research show that 7,8-disubstituted purine-2,6-dione derivatives represent a group of pan-PDE inhibitors with high inhibitory potency against individual PDE isoenzymes. They can effectively and strongly inhibit many different PDEs, including those relevant to the pathogenesis of respiratory diseases [1,3]. Recently, we confirmed 145 favorable binding modes in the active site of PDE4B and PDE7A as well as to the TRPA1 model [20]. Moreover, molecular docking studies of close analogs of compounds 832 and 869, which recognized crucial interactions in PDE4B/PDE7A and TRPA1 sites, respectively, were previously described [20,21]. Here, it was demonstrated for the first time that these potent, pan-PDE inhibitors exert a strong limiting effect on TGF-β_1_-induced FMT in lung fibroblasts. Selected 7,8-disubstituted purine-2,6-dione derivatives, especially 145 inhibited the expression of genes and proteins characteristic for both myofibroblasts and extracellular matrix proteins secreted by these cells. We showed that some compounds belonging to 7,8-disubstituted purine-2,6-dione derivatives can also reduce proliferation, migration, and contraction of pulmonary fibroblasts. We also displayed that the observed anti-fibrotic effects of pan-PDE inhibitor, 145, are the result of its impact on the canonical TGF-β signaling pathway. This compound, decreased Smad-2 phosphorylation and subsequent translocation to the nucleus, thereby reducing TGF-β_1_-induced and Smad-mediated transcription of profibrotic genes in lung fibroblasts (Figure 8).

The Smad-dependent TGF-β signaling pathway has already been identified as a potential therapeutic target for preventing fibrosis in the lung and other organs [36,37]. This has been confirmed by our current and previous data, in which we have also shown that in asthmatic fibroblasts, pentoxifylline and lisofylline can limit TGF-β signaling [33]. Further evidence is provided by Fehrholz et al. who demonstrated in lung epithelial cells that caffeine and rolipram might also affect Smad-dependent TGF-β signaling [38]. Similar effects have been described for the new drugs used in idiopathic pulmonary fibrosis (IPF) therapy, pirfenidone, and nintedanib [39]. Although the exact mechanism of action of these anti-inflammatory, antioxidant and anti-remodeling drugs is yet unknown, it is has been found that they can affect, among others, TGF-β/Smad-dependent signaling [39,40].

Some studies revealed that increased intracellular cAMP levels could directly affect the canonical TGF-β and Smad-dependent signaling pathway [41,42,43,44,45]. After an increased CREB phosphorylation (e.g., in response to the elevated intracellular cAMP levels), a CREB-binding protein (CBP) effectively binds p-CREB resulting in enhancement of its transcriptional activity [46]. On the other hand, it is known that CBP can directly interact with the Smad protein complex and operate as a Smad transcriptional co-activator [47]. As such, competition between cAMP-dependent CREB/CBP binding and TGF-β-dependent Smad/CBP binding for the common co-activator CBP [43,45] is proposed as the mechanistic explanation for the functional inhibition of Smad-dependent signaling. Compound 145, as a strong PDE inhibitor, elevated cAMP levels in lung fibroblasts, activated PKA and, as a consequence, led to increased CREB phosphorylation, which in turn inhibited Smad signaling (Figure 8). The presence of 145 caused the most prominent activation of the cAMP/PKA/CREB pathway in TGF-β_1_-induced MRC-5 cells. At the same time, this compound had the best inhibitory effect on TGF-β_1_/Smad-2 signaling. While a strong activation of cAMP-dependent CREB phosphorylation is not surprising given that 145 elevates intracellular cAMP, its inhibitory effect on Smad-2 phosphorylation indicates new and desirable properties of this compound (Figure 8). Since other Smad proteins can also regulate TGF-β signaling, the effect of 145 on Smad-3/4 or Smad-1 should also be tested in future studies. The increased efficacy of compound 145 may be the result of the interaction with two independent signaling pathways, both of which promote anti-fibrotic effects. It is known that the reduction of TGF-β-induced and Smad-mediated transcription of profibrotic genes is one of the main goals of anti-fibrotic therapy [36,37] and out of the studied compounds, 145 best meets this requirement. Of note, is that there are also other reports that associate CREB phosphorylation and activation of CREB-mediated transcription with anti-inflammatory properties, e.g., increased IL-10 synthesis, inhibition of NF-κB signaling, Th17 cell differentiation, and survival of T_reg_ cells [48,49]. Therefore, it is likely that the high CREB phosphorylation induced by 145, compared to other tested compounds, may explain the prominent anti-fibrotic effects of this compound in TGF-β_1_-induced MRC-5.

We have characterized compounds 832 and 145 as strong, pan-PDE inhibitors. However, despite the similar affinity of both compounds to, e.g., PDE1, PDE3, or PDE4, only 145 was able to inhibit, e.g., PDE4D, PDE5A, or PDE8A. Compound 145 ability to inhibit PDE8A is an important feature because even the currently known non-selective inhibitors, such as IBMX (also used in this study), are unable to inhibit PDE8, probably due to lack of the interaction in the binding site, i.e., hydrogen bond with Tyr748 [50]. The literature data showed that PDE8 significantly differs from other PDE isoenzymes [50]. Recent reports indicate that this isoform may be of particular importance for the development of effective PDE inhibitors [4,51]. Both the elevated cAMP levels in MRC-5 treated with 145, as well as the increased anti-fibrotic properties of this compound, may result from its interaction with PDE8. Another reason for enhanced 145 activity may be its ability to inhibit cGMP-specific, PDE5A. This also suggests a possible involvement of cGMP in 145 anti-fibrotic effects in MRC-5 cells. In turn, 145 PDE4D and PDE3 inhibition may result in inducing nausea and ionotropic effects, respectively. On the other hand, the potential side-effects caused by pan-PDE inhibitors can be mitigated by reducing their potential therapeutic dose, while maintaining a similar impact to selective PDE inhibitors.

Previous studies [20] made us aware that 145 may also interact with TRPA1 channels. In light of the reports indicating that TRPA1 channels may be a potential molecular target for lung diseases [18,52,53], and since caffeine can suppress TRPA1 activity [54], we examined whether 832, 869, and 145 affect the function of this channel. Our results do not support a very strong agonistic/antagonistic activity of 145 towards TRPA1, but functional analysis revealed that this compound can modify calcium influx in pulmonary fibroblasts in a way a weak agonist would. To assess the involvement of TRPA1 in the observed activity of compound 145, we performed a series of experiments in which we pharmacologically blocked or activated this ion channel and then compared the effects in the presence and absence of 145. The presence of 145 neither affected MRC-5 proliferation or cAMP levels, which indicates that the TRPA1 component may not play an important role in 145-mediated anti-fibrotic effects. We cannot exclude that 145 exerts such a strong inhibitory effect on PDE that the maximum anti-fibrotic potential is already achieved, and thus, any further modulation of TRPA1 activity may be negligible for the final outcome. The slight but noticeable effect of the TRPA1 antagonists—HC-030031 and 869, on some tested TGF-β_1_-induced MRC-5 myofibroblast features associated with remodeling may support this notion. This is also in line with several clinical trials in which TRPA1 antagonists are tested to treat respiratory diseases [53]. On the other hand, Kurahara et al. demonstrated that pirfenidone might exert anti-inflammatory and anti-fibrotic effects upon TRPA1 activation [55]. We have not seen similar effects in our study; in fact, TRPA1 activation via ASP 7663 did not result in significant changes in the phenotype of TGF-β_1_-induced MRC-5 cells. Considering the above reports, and since large and sustained intracellular Ca^2+^ elevations are often noxious for cells and may trigger cell death [56], the fact that compound 145 does not excessively activate Ca^2+^ influx via TRPA1 might be desirable from the therapeutic perspective. Collectively, it could be argued that anti-fibrotic properties of compound 145 are accomplished by a strong PDE inhibitor activity, while the TRPA1 channel modulation is minor and may only slightly contribute to 145 activity.

One of the further mechanisms of methylxanthine interaction with its cellular targets is that these compounds may function as adenosine receptor antagonists. Our earlier studies carried out on airway smooth muscle cells did not support the concept of adenosine A_1_ or A_2A_ receptor antagonism as a potent modulator of TGF-β-induced airway remodeling [19]. In turn, Roberts et al. showed that selected agonists of the Gs-coupled receptors, including, e.g., adenosine A_2B_ or prostacyclin receptors, can inhibit lung fibroblast differentiation into myofibroblasts [57]. The effect of these compounds was comparable to that achieved after administration of the adenylate cyclase activator, forskolin, but it was not consistently associated with the level of cAMP [57]. Here, we demonstrate a link between PDE inhibition, increased cAMP levels, activation of the PKA/CREB pathway, and anti-fibrotic properties for both, compound 145 and forskolin. Considering the results of SAR studies indicating which chemical substituents predispose a compound to possess adenosine receptor antagonist activity [58], and our earlier data indicating a lack of effect of adenosine antagonists on TGF-β_1_-induced remodeling in ASMC, we believe that adenosine antagonistic effects do not contribute to the observed effects of the 7,8-disubstituted purine-2,6-dione derivatives in our study. Calcium signals have also been implicated in the mechanism of action of methylxanthine compounds [59,60]. Disturbances in calcium homeostasis may also lead to the synthesis of the extracellular matrix and other profibrotic proteins in pulmonary fibroblasts [56]. 7,8-disubstituted purine-2,6-dione derivatives turned out to have poor activity towards calcium-dependent TRPA1 channels.

Our previous research demonstrated that compounds from the group of 7,8-disubstituted purine-2,6-dione derivatives are able to limit ASMC remodeling response, and here we confirm 145 preferential activity in reducing lung fibroblast features associated with remodeling. Although both the previous and current studies provide solid evidence of the anti-fibrotic effect of compound 145, the studies so far have been performed in vitro in the presence of one growth factor. Therefore, the activity of 145, a new pan-PDE inhibitor, should be verified in vivo in a model of chronic lung diseases and compared with known anti-inflammatory drugs or PDE inhibitors already used clinically. In the in vivo model, it would be possible to know the 145 pleiotropic effects. Given the complexity of bronchial and lung remodeling, the search for compounds with a wide spectrum of activity is extremely valuable. Considering the data obtained with ASMCs and presented here lung fibroblasts, it can be concluded that the group of 7,8-disubstituted purine-2,6-dione derivatives offers promising drug candidates targeting chronic lung disease therapy.

## 4. Materials and Methods

### 4.1. Tested Compounds

*N*′-(2,4-dihydroxybenzylidene)-4-(8-((furan-2-ylmethyl)amino)-1,3-dimethyl-2,6-dioxo-1,2,3,6-tetrahydro-7*H*-purin-7-yl)butanehydrazide (working name 832, described in [21]), *N*-(4-(*tert*-butyl) phenyl)-2-(8-methoxy-1,3-dimethyl-2,6-dioxo-1,2,3,6-tetrahydro-7*H*-purin-7-yl)acetamide (working name 869, described in [20]), and 4-(8-butoxy-1,3-dimethyl-2,6-dioxo-1,2,3,6-tetrahydro -7*H*-purin-7-yl)-*N*-(5-(*tert*-butyl)-2-hydroxyphenyl)butanamide (working name 145, described in [20]) were used in this study. These compounds were synthesized according to a multistep procedure, as described previously [20,21]. Briefly, to obtain 832, 8-bromo-1,3-dimethyl-3,7-dihydro-1*H*-purine-2,6-dione was treated with furan-2-ylmethanamine in refluxing 2-methoxyethan-1-ol. Next, the 8-[(furan-2-yl)methyl]amine derivative was alkylated at position 7 using ethyl 4-bromobutanoate in the presence of K_2_CO_3_ and a catalytic amount of *N*-benzyl-*N*,*N*-diethylethanaminium chloride (TEBA) in refluxing acetone. Treatment of the obtained ester with hydrazine hydrate in anhydrous ethanol gave the corresponding hydrazide. In the final step, the hydrazide was condensed with 2,4-dihydroxybenzaldehyde in methanol in the presence of a catalytic amount of HCl. To obtain 869, 8-bromo-1,3-dimethyl-3,7-dihydro-1*H*-purine-2,6-dione was alkylated at position 7 with ethyl 2-chloroacetate in the presence of K_2_CO_3_ and a catalytic amount of TEBA in refluxing acetone. The synthesized ethyl 2-(8-bromo-1,3-dimethyl-2,6-dioxo-1,2,3,6-tetrahydro-7*H*-purin-7-yl)acetate was treated with sodium methanolate in methanol, then hydrolyzed using potassium hydroxide in a water-acetone mixture and acidified with concentrated HCl to obtain the corresponding acid. In the final step, the obtained acid was condensed with 4-*tert*-butylaniline in *N,N*-dimethylformamide in the presence of di(1*H*-imidazol-1-yl)methanone. 145 was obtained in a similar manner, but ethyl 4-bromobutanoate was used for the alkylation, the transesterification reaction was carried out using sodium butanolate in butanol, and in the final stage, the resulting acid was condensed with 2-amino-4-(*tert*-butyl)phenol [20]. Reference substances used in the studies were: 3-isobutyl-1-methyl-3,7-dihydro-1*H*-purine-2,6-dione (IBMX, Sigma Aldrich, St. Louis, MO, USA), 2-(1,3-dimethyl-2,6-dioxo-1,2,3,6-tetrahydro-7*H*-purin-7-yl)-*N*-(4-isopropylphenyl)acetamide (HC-030031, Sigma Aldrich, St. Louis, MO, USA) and (*E*)-2-(7-fluoro-1-isobutyl-2-oxoindolin-3-ylidene)acetic acid (ASP 7663, Sigma Aldrich, St. Louis, MO, USA). In some experiments, the adenylyl cyclase activator, forskolin (Sigma Aldrich, St. Louis, MO, USA) was used. All evaluated compounds were dissolved in dimethylsulfoxide (DMSO, PAN-Biotech, Germany) and diluted in culture medium (the final DMSO concentration did not exceed 0.5% and was not harmful to the cells).

### 4.2. PDE Subtype Selectivity

Inhibitory activities of the investigated compounds against various subtypes of human recombinant (hr)PDEs, namely PDE1A, PDE1B, PDE1C, PDE3A, PDE3B, PDE4A, PDE4B, PDE4D, PDE7A, and PDE8A were assessed using a PDE-Glo^TM^ Assay Kit (Promega, Madison, WI, USA) according to the protocol provided by the manufacturer. Briefly, appropriate amounts of each enzyme (SignalChem, Richmond, B.C., Canada), diluted with PDE-Glo reaction buffer, were distributed on a 384-well plate (Thermo Scientific, Waltham, MA, USA). The investigated compounds were dissolved in DMSO, diluted with the same vehicle, and mixed with the reaction buffer at a *v/v* ratio of 1:5. Thereafter, 1 µL of these mixtures and 2.5 µL of the cAMP solution as a substrate were added. The final cAMP concentration was 0.05 µM. The samples were incubated for 10 min at 30 °C. Then, 2.5 µL of termination buffer and 2.5 µL of PDE-Glo detection solution were added to each well and incubated for 20 min. Finally, 10 µL of PDE-Glo kinase reagent was added, and luminescence was measured using the POLARstar Omega plate reader (BMG LABTECH, Ortenberg, Germany). Each sample was prepared in quadruplicate. The relative activities (A, %) of all samples were calculated from the following equation: A = [(LU_sample_ − LU_blank_)/(LU_control_ − LU_blank_)]·100%; where LU_control_ is the luminescence of a control sample in the absence of a PDE inhibitor, LU_sample_ is a luminescence of an investigated sample in the presence of an inhibitor, and LU_blank_ is a luminescence of a sample in the absence of enzyme (activity = 0). IC_50_ values were estimated using non-linear regression.

### 4.3. TRPA1 Assay

The TRPA1 agonist and antagonist fluorescent imaging plate reader (FLIPR) Assay (functional, HEK293 cell-based) was performed at Eurofins Panlabs, Inc. (St. Charles, MO, USA). The compounds were prepared in assay buffer to the final dilutions 10 and 50 µM. In each experiment, the respective reference compounds were tested: allyl isothiocyanate (the agonist assay) and ruthenium red (the antagonist assay). The electrophysiological assays were conducted on a FLIPR^TETRA^ instrument where the test compounds, vehicle controls, and reference agonists or antagonists were added to the assay plate after a fluorescence baseline was established. The agonist or antagonist incubation during the assay was for a total of 180 s and was used to assess each compound’s ability to activate or inactivate TRPA1. All plates were subjected to appropriate baseline corrections. Once baseline corrections were processed, maximum fluorescence values were exported, and data was manipulated to calculate percentage activation or percentage inhibition. Results showing activity greater than 25% were considered to represent significant effects of test compounds. Experiments were run three times, and at least 20 individual cells were recorded in each experiment.

### 4.4. In Vitro Lung Fibroblast Culture

The normal human lung fibroblast cell line—MRC-5 (ATCC^®^ CCL-171™, Manassas, VA, USA)—was used in the study. Cells were cultured in Eagle’s Minimum Essential Medium (EMEM, ATCC^®^ 30-2003™, Manassas, VA, USA) supplemented with 10% (*v/v*) fetal bovine serum (FBS; Gibco, Thermo Fisher Scientific, Waltham, MA, USA) and antibiotics mixture (penicillin, streptomycin, amphotericin B; Gibco, Thermo Fisher Scientific, Waltham, MA, USA) (standard culture medium) in standard culture conditions (5% CO_2_, 37 °C, 95% humidity). Unless otherwise specified, for the experiments, cells were seeded at a final density (5 × 10^3^/cm^2^) in standard culture medium for 24 h and then cultured in EMEM at reduced serum content (0.5%; *v/v*) for additional 24 h. TGF-β_1_ (BD Biosciences, San Jose, CA, USA) was administered at 5 ng/mL concentration in all experiments.

### 4.5. Viability and Proliferation Assays

MRC-5 cell viability was evaluated using CytoTox-ONE™ Homogeneous Membrane Integrity Assay (Promega Corporation, Madison, WI, USA) according to the manufacturer’s protocol. The average fluorescence intensity with an excitation wavelength of 560 nm and an emission wavelength of 590 nm was measured using a microplate reader (SpectraMax^®^ iD3, Molecular Devices, San Jose, CA, USA). Experiments were run three times in duplicates. MRC-5 proliferation was determined using the alamarBlue^®^ assay. Cells were seeded for 24 h in standard culture medium, then serum-starved for an additional three days. Evaluated compounds (1-50 µM) were added 1 h prior FBS (10%, *v/v*) for an additional 48 h. After incubation, the alamarBlue^®^ reagent (Invitrogen, Life Technologies, Carlsbad, CA, USA), dissolved in Hank’s Balanced Salt Solution (10% *v/v*; Gibco, Thermo Fisher Scientific, Waltham, MA, USA), was added for 30 min. After the observed color change, the supernatant was transferred to 96-well plates, and the absorbance was measured at 570 and 600 nm. The percent difference in reduction between treated and control cells was calculated according to the manufacturer’s protocol. The experiments were run three times in duplicates.

### 4.6. Transwell Migration Assay

MRC-5 migration was evaluated using 6.5 mm Transwell culture plates with an 8.0 µm pore polycarbonate membrane (Corning Incorporated, NY, USA). Cells were serum-deprived for 24 h, trypsinized and seeded in the upper chamber in serum-free culture medium supplemented with or without study compounds (10 µM). The lower compartment was filled with the same culture medium, and after 1 h TGF-β_1_ was added to the wells. After 24 h of incubation cells were fixed with 4% formaldehyde solution (Sigma Aldrich, St. Louis, MO, USA) and stained with 0.5% crystal violet solution (Sigma Aldrich, St. Louis, MO, USA). The non-migrated cells were removed from the upper face of the membranes using a cotton swab. The number of migrated cells was counted under an inverted microscope (Nikon Eclipse TS 100, 10× objective) using 10 randomly selected fields of view. Experiments were run twice, in a blind-folded manner.

### 4.7. Cell Contraction Assay

A collagen cell contraction assay was performed according to manufactures’ instructions (Cell Biolabs, INC., San Diego, CA, USA). Briefly, MRC-5 was harvested and mixed with a collagen solution. After polymerization, collagen gel lattices were cultured for two days, during which mechanical tension develops. Before releasing the stressed matrix, MRC-5 cells were pre-treated with study compounds (10 µM) for 1 h. Then collagen gels were gently released from the sides of the culture dishes and contraction in the presence of study compounds, and TGF-β_1_ was initiated. The collagen gel size was monitored at various time points and quantified with the Fiji ImageJ Software (National Institutes of Health, Bethesda, MD, USA, version 1.52q). The experiments were run three times.

### 4.8. Intracellular Calcium Measurements

MRC-5 cells were briefly washed with NaHEPES buffer (NaHEPES buffer was prepared as follows: NaCl 140 mM, KCl 4.7 mM, HEPES 10 mM, MgCl_2_ 1 mM, glucose 10 mM; pH 7.3) and then loaded with 1 µM Fluo-4 AM (Thermo Fisher Scientific, Waltham, MA, USA) for 1 h, 37 °C in NaHEPES. After the incubation, the cells were washed again with fresh NaHEPES and used for experiments in a flow chamber perfused with NaHEPES-based extracellular solution supplemented with 1 mM CaCl_2_. All experiments were carried out at room temperature using Zeiss LSM 880 confocal microscope (40× oil objective), excitation was set to 488 nm, and the emission was between 500 and 600 nm. Each series of images was recorded at 256 × 256-pixel resolution, two consecutive frames were averaged, and the interval between the averaged images was set at 2 s. Fluorescence signals were plotted as F/F_0_, where F_0_ was baseline fluorescence defined as the averaged signal from ten consecutive baseline images.

### 4.9. cAMP ELISA

MRC-5 cells were seeded in standard culture medium for 24 h and then serum-starved for 2 h in HBSS. The study compounds (10 µM) were added to the MRC-5 cultures and after 30 min of incubation, the intracellular cAMP level in cell lysates was measured using a colorimetric cAMP ELISA Kit according to the manufacturer’s protocol (Cell Biolabs, INC., San Diego, CA, USA). Obtained results were normalized to total protein content in each sample. The experiments were run two times in duplicates.

### 4.10. In-Cell ELISA

The relative α-SMA and collagen I level in MRC-5 cells were measured using an in-cell ELISA assay. Study compounds (10 µM) were added 1 h before TGF-β_1_ treatment, and the content of both proteins in fibroblasts was determined after 48 h of incubation. Briefly, MRC-5 were fixed in methanol, permeabilized and blocked in 1% bovine serum albumin (BSA) with 0.1% Tween^®^ 20 and then immunostained with primary mouse, monoclonal anti-α-SMA or anti-collagen type I antibodies (#A2547 or #SAB4200678, respectively, Sigma Aldrich, St. Louis, MO, USA), followed by secondary, anti-mouse, peroxidase-conjugated antibody (#A9044, Sigma Aldrich, St. Louis, MO, USA). The colorimetric reaction resulting from the addition of peroxidase substrate was stopped, and the absorbance was measured at 450 nm. The relative protein amount was normalized to the total cell number in each sample (Janus Green staining). The experiments were run four times in duplicates.

### 4.11. Immunofluorescence Labelling

For immunocytochemical labeling, MRC-5 cells were seeded on glass coverslips. Study compounds (10 µM) were added 1 h before TGF-β_1_ treatment, and analysis was performed after 1 h (p-Smad-2 and p-CREB detection) or 48 h (α-SMA detection) of incubation. Cells were fixed in methanol or 4% formaldehyde solution, permeabilized in 0.2% or 0.1% Triton X-100 solution (for p-Smad-2 and p-CREB or α-SMA, respectively). After incubation in blocking solution the following primary antibodies: rabbit polyclonal, anti-phospho-Smad-2 (Ser465, Ser467) antibody (#44-244G, Thermo Fisher Scientific, Waltham, MA, USA); rabbit polyclonal, anti-phospho-CREB1 (Ser133) antibody (#orb213775, Biorbyt, Cambridge, United Kingdom); mouse monoclonal, anti-α-SMA antibody (#MA5-11547, Thermo Fisher Scientific, Waltham, MA, USA) and secondary antibodies: goat anti-rabbit Alexa Fluor 546 conjugated antibody and goat anti-mouse Alexa Fluor 488 conjugated antibody (#A-11010 and #A-11001, respectively, Invitrogen, Carlsbad, CA, USA) were used. Nuclei were counterstained with Hoechst 33342 dye (Thermo Fisher Scientific, Waltham, MA, USA). Slides were mounted in ProLong™ Glass Antifade Mountant (Invitrogen, Carlsbad, CA, USA) and analyzed using Leica DMiL LED Fluo microscope (for α-SMA and p-CREB visualization, 40× objective) or Leica DMi8 with Thunder Imaging System (for p-Smad-2 visualization, 40× objective), all equipped with LAS-X Software (Leica Microsystems GmbH, Wetzlar, Germany). Experiments were run four times using five randomly selected fields of view, at the same fluorescent time exposure and in a blind-folded manner.

### 4.12. Quantitative PCR

Quantitative polymerase chain reaction (qPCR) was performed to assess the expression level of selected human genes: *ACTA2, MYH11, SM22, COL1A1, TNC, FN1,* and *VCAN*. MRC-5 cells were preincubated with study compounds (10 µM) for 1 h, followed by 24 h incubation with TGF-β_1_, and then total RNA was extracted using Total RNA Mini Kit (A&A Biotechnology, Gdynia, Poland) according to the manufacturer’s protocol. RNA concentration was measured with a BioSpectrometer^®^ basic (Eppendorf), and equal amounts of total RNA (about 1 µg) were reverse-transcribed using the iScript™ cDNA Synthesis Kit (BioRad, Hercules, CA, USA). qPCR assays were performed using the CFX96 Touch Real-Time PCR Detection System (BioRad, Hercules, CA, USA) and SsoAdvanced^TM^ Universal SYBR^®^ Green Supermix (BioRad, Hercules, CA, USA). qPCR cycling was performed with denaturation at 94 °C for 30 s, annealing at 59 °C for 30 s and an extension at 72 °C for 30 s. Specific primers used to quantify the transcripts are listed in Table 3. The relative abundance of specific mRNA transcripts was estimated based on cycle threshold (Ct) value and recalculated against the endogenous reference 18S ribosomal RNA gene (*18S rRNA*) using the ∆Ct method. The experiments were run three times in duplicates.

### 4.13. Western Blotting

Western Blot analysis was performed according to a standard protocol. Briefly, MRC-5 cells were pre-incubated with the study compounds (10 µM) for 1 h, followed by 48 h incubation with TGF-β_1_ and then lysed in RIPA buffer (RIPA Lysis and Extraction Buffer, Thermo Fisher Scientific, Waltham, MA, USA) and supplemented with protease inhibitor cocktail and phosphatase inhibitor cocktail (both from Sigma Aldrich, St. Louis, MO, USA). Equal amounts of protein (30 µg) were separated using 10% SDS-polyacrylamide gels and transferred onto polyvinylidene difluoride (PVDF) membranes (Thermo Fisher Scientific, Waltham, MA, USA). Then membranes were washed with Tris Buffered Saline with Tween 20 (TBST) buffer (150 mM NaCl, 50 mM Tris, 0.05% Tween 20), blocked with 5% skim milk in TBST and incubated overnight with the appropriate primary antibody: rabbit, polyclonal anti-SMAD2 antibody, dilution 1:250, #51-1300 and rabbit, polyclonal anti-phospho-SMAD2 (Ser465, Ser467) antibody, dilution 1:500, #44-244G (both from Thermo Fisher Scientific, Waltham, MA, USA); rabbit, polyclonal anti-CREB1 antibody, dilution 1:500, #orb213777 and rabbit, polyclonal anti-phospho-CREB1 (Ser133) antibody, dilution 1:500, #orb213775 (both from Biorbyt LLC, San Francisco, CA, USA); mouse monoclonal anti-GAPDH antibody, dilution 1:5000, #G8795 (Sigma Aldrich, St. Louis, MO, USA). The next day, after gentle rising, membranes were exposed to secondary, rabbit anti-mouse IgG (whole molecule) peroxidase antibody (#A9044, dilution 1:3000, Sigma Aldrich, St. Louis, MO, USA) and goat anti-rabbit IgG (H+L) peroxidase-conjugated antibody (#32460, dilution 1:3000, Thermo Fisher Scientific, Waltham, MA, USA). Blots were visualized using a chemiluminescence method (Clarity Max™ Western ECL Substrate—Peroxide Solution, BioRad, Hercules, CA, USA) with a C-DiGit^®^ Blot Scanner (LI-COR Biosciences, Lincoln, NE, USA). ROD was measured according to the NIH guidelines with Fiji ImageJ Software. The experiments were run four times.

### 4.14. Protein Kinase A Activity

Protein Kinase A (PKA) activity in MRC-5 cells was determined using a PKA Colorimetric Activity Kit (Invitrogen, Carlsbad, CA, USA) according to the manufacturer’s protocol. MRC-5 cells were seeded in standard culture medium, and after 48 h they were serum-starved for 2 h in HBSS, followed by the addition of study compounds (10 µM) and incubation for 30 min. Finally, TGF-β_1_ was administered, and the samples were incubated for an additional 30 min. Whole cell lysates were used in the assay. The amount of PKA was normalized to the total protein content in each sample. The experiments were run two times in duplicates.

### 4.15. Statistical Analysis

Statistical analysis was performed with GraphPad Prism (GraphPad Software, Inc., San Diego, CA, USA, version 5.01). The comparisons between experimental conditions were performed using a non-parametric Wilcoxon test for paired data and a non-parametric Mann–Whitney U test (for qPCR data). Values presented in the graphs correspond to the mean ± standard error of the mean (S.E.M.). *P* < 0.05 was considered statistically significant.

## Figures and Tables

**Table 1:** Compounds 832, 869, and 145 as PDE inhibitors.

Compound	PDE-IC_50_ [µM]										
	1A	1B	1C	3A	3B	4A	4B	4D	5A	7A	8A
832	7.24	0.04	89.18	0.125	0.42	4.75	4.4 ^3^	n.a.	n.a.	10.5 ^3^	n.a.
869	n.a.	n.a.	172.18	n.a.	156.70	58.05	n.a.	n.a.	n.a.	n.a.	n.a.
145	n.a.	2.69 ^1^	48.52	0.18 ^1^	2.05	1.59	5.43 ^2^	13.85 ^1^	103.88 ^1^	5.07 ^2^	73.18
IBMX	0.11	35.70 ^1^	12.52	0.71 ^1^	0.31	16.39	46.60 ^4^	81.94	45.09 ^1^	77.70 ^4^	n.a.

Values published previously: ^1^ [19]; ^2^ [20]; ^3^ [21]; ^4^ [22]; n.a.—not active

**Table 2:** Compounds 832, 869, and 145 as TRPA1 modulators.

Compound	TRPA1 Inhibition (% of control)		TRPA1 Activation (% of Control)	
	10 µM	50 µM	10 µM	50 µM
832	12.4	8.8	3.8	2.4
869	74.9	98.7	0.0	2.2
145	12.3	4.8	0.2	4.6
HC-030031	91.8	98.8	-	-
ASP 7663	-	-	75.8	76.1

**Table 3:** Sequences of primers used.

Gene	Forward Sequence (5′→3′)	Reverse Sequence (5′→3′)
*ACTA2*	CCGGGAGAAAATGACTCAAA	GAAGGAATAGCCACGCTCAG
*MYH11*	CAGATGCTGGACCTTGAAGA	TCCATGACCAGGATCTCATC
*SM22*	CGCGAAGTGCAGTCCAAAATCG	GGGCTGGTTCTTCTTCAATGGGC
*COL1A1*	AGCCAGCAGATCGAGAACAT	TCTTGTCCTTGGGGTTCTTG
*TNC*	CCAGCGACCATCAACGCAGC	GGGGCTTGTTCAGTGGATGCCT
*FN1*	TCGAGGAGGAAATTCCAATG	ACACACGTCCACCTCATCAT
*VCAN*	GGGAACCTGGTGAAGAAACA	CTTCCACAGTGGGTGGTCTT
*18S rRNA*	CGCCGCTAGAGGTGAAATTC	TTGGCAAATGCTTTCGCTC

Abbreviations: *ACTA2*—actin alpha 2, *MYH11*—myosin-heavy chain 11, *SM22*—transgelin, *COL1A1*—collagen type I alpha 1 chain, *TNC*—tenascin C, *FN1*—fibronectin 1, *VCAN*—versican.

## Supplementary Materials

The following are available online at https://www.mdpi.com/1422-0067/21/11/4008/s1, Table S1: MRC-5 proliferation in the growing concentrations of studied compounds. Values represent data presented on Figure 2B and Table S2: MRC-5 proliferation in the growing concentrations of studied compounds. Values represent data presented on Figure 5B.
