# Supplementary material for: A Novel, Pan-PDE Inhibitor Exerts Anti-Fibrotic Effects in Human Lung Fibroblasts via Inhibition of TGF-β Signaling and Activation of cAMP/PKA Signaling
Source: Int J Mol Sci. 2020 Jun 3;21(11):4008. doi: 10.3390/ijms21114008 (PMC7312375; doi:10.3390/ijms21114008)
Supplement: Supplementary file 1 [file ijms-21-04008-s001.pdf]

International Journal of *Molecular Sciences*

MDPI

Supplementary Materials: A Novel, Pan-PDE Inhibitor Exerts Anti-Fibrotic Effects in Human Lung Fibroblasts via Inhibition of TGF-β Signaling and Activation of cAMP/PKA Signaling

Katarzyna Wójcik-Pszczoła, Grażyna Chłoń-Rzepa, Agnieszka Jankowska, Marietta Ślusarczyk, Paweł E Ferdek, Agnieszka A Kusiak, Artur Świerczek, Krzysztof Pociecha, Paulina Koczurkiewicz-Adamczyk, Elżbieta Wyska, Elżbieta Pękala and Reinoud Gosens

Table S1. MRC-5 proliferation in the growing concentrations of studied compounds. Values represent data presented on Figure 2B

Compound concentration [µM]	MRC-5 proliferation [% of control]					
	832	869	145	IBMX	HC-030031	ASP 7663
**1**	93	91	104	102	98	99
**5**	87	89	91	99	96	97
**10**	85	90	84	98	95	96
**15**	82	88	74	100	90	92
**20**	83	87	72	95	90	89
**50**	61	75	42	90	85	86

Table S2. MRC-5 proliferation in the growing concentrations of studied compounds. Values represent data presented on Figure 5B

Compound concentration [µM]	MRC-5 proliferation [% of control]		
	145	HC-030031 + 145	ASP 7663 + 145
**1**	93	96	94
**5**	84	93	93
**10**	83	80	86
**50**	46	56	60
